# Genomes in clinical care

**DOI:** 10.1038/s41525-024-00402-2

**Published:** 2024-03-14

**Authors:** Olaf Riess, Marc Sturm, Benita Menden, Alexandra Liebmann, German Demidov, Dennis Witt, Nicolas Casadei, Jakob Admard, Leon Schütz, Stephan Ossowski, Stacie Taylor, Sven Schaffer, Christopher Schroeder, Andreas Dufke, Tobias Haack

**Affiliations:** 1 Institute of Medical Genetics and Applied Genomics, University of Tübingen, Tübingen, Germany; 2 NGS Competence Center Tübingen, University of Tübingen, Tübingen, Germany; 3 Center for Rare Diseases Tübingen, University of Tübingen, Tübingen, Germany; 4 Institute for Bioinformatics and Medical Informatics (IBMI), University of Tübingen, Tübingen, Germany; 5 Illumina, Inc, San Diego, CA USA

**Keywords:** Medical genetics, DNA sequencing, Medical genomics, RNA sequencing, Genetic testing

## Abstract

In the era of precision medicine, genome sequencing (GS) has become more affordable and the importance of genomics and multi-omics in clinical care is increasingly being recognized. However, how to scale and effectively implement GS on an institutional level remains a challenge for many. Here, we present Genome First and Ge-Med, two clinical implementation studies focused on identifying the key pillars and processes that are required to make routine GS and predictive genomics a reality in the clinical setting. We describe our experience and lessons learned for a variety of topics including test logistics, patient care processes, data reporting, and infrastructure. Our model of providing clinical care and comprehensive genomic analysis from a single source may be used by other centers with a similar structure to facilitate the implementation of omics-based personalized health concepts in medicine.

## Introduction

Genome sequencing (GS) is transforming how we diagnose, treat, and manage a significant number of genetic conditions including rare diseases and cancer. Due to constantly growing high-throughput sequencing and data analysis capacities and falling costs, the application of GS is broadening as a first-line diagnostic in routine clinical care. Importantly, data analysis of the genome also allows us to go beyond that of diagnosing the underlying disease cause. For example, genome analysis can enable the identification of monogenic and polygenic risks for not yet manifested diseases (i.e., genetic predictive diagnostics).

### Why genomes

**Fig. 1:**
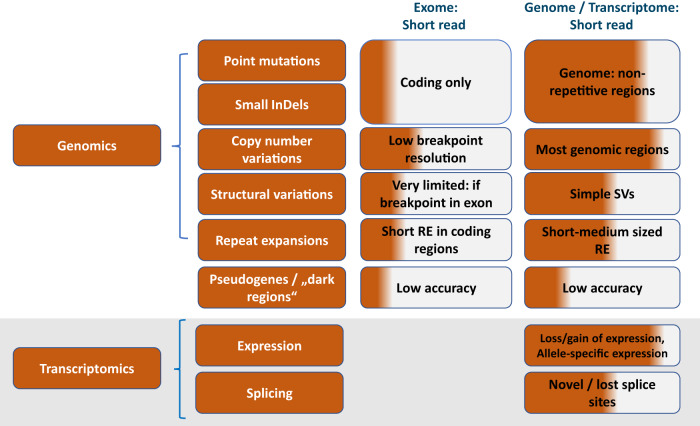
Diagnostic power of srGS compared to ES. Different areas of application are shown (genomics, transcriptomics, and epigenomics). The shade of the color represents the coverage/potential detection rate of the respective variants/genome/transcriptome regions.

The primary goal of bringing GS into routine clinical care is to provide a precise molecular diagnosis to patients with rare disease and familial cancer, in the hope that such knowledge can alter medical management and improve patient outcomes. Current standard of care testing for genetic disorders includes Sanger sequencing, chromosomal microarray, targeted sequencing panels, and exome sequencing (ES). However, a growing body of evidence has demonstrated that GS has technical advantages and diagnostic efficiency over that of other next-generation sequencing (NGS) tests (Fig. 1). First of all, ES does not mean “whole exome” as the enrichment process it does not cover all genes or all exonic regions. In particular, GC-rich exons commonly present in the first exons of most genes are poorly enriched. Also, while copy number variations (CNVs), inversions, and numerous repeat expansions can be missed by ES analysis, GS enables their detection with high sensitivity and specificity (in terms of integration site or even deletion breakpoints)^1–4^. We have encountered several patients with neurological disorders caused by repeat expansions with a broader phenotype than is described in the literature analyzing genome data^5^. In addition, the number of repeat expansion disorders is growing steadily making it challenging and costly to rule out all potentially disease-causing repeats by single targeted analyses. Nevertheless, in the field of repeat expansions, the currently used short-read NGS technology has its limitations as well as it will miss very long repeat expansions and for some diseases, as described for CANVAS, repeat composition is crucial to define pathogenetic relevance of the repeat expansion^6^. Also, epigenetic diseases may be missed by short-read GS but could potentially be detected by long-read GS (lrGS). However, the clinical utility of lrGS to diagnostically define disease-causing epigenetic alterations and repeat expansions needs still to be demonstrated.

The technological advantages of GS translate into clinical advantages. Because GS detects nearly all forms of disease-causing variation, it has a higher chance of providing a precise molecular diagnosis to patients that can inform clinical care. Studies have shown that GS can initiate a cascade of health outcome-altering events such as changes in pharmacotherapy, referral to specialists, avoidance of unnecessary procedures, and stoppage of ineffective treatments^7–10^. In fact, a recent meta-analysis on the clinical utility of GS and ES found that the former resulted in significantly more changes in management compared to the latter^11^.

To enable the widespread adoption of GS into routine clinical care, it is important to develop an understanding of how to effectively implement and scale the testing on an institutional level. Such factors include automation, turnaround time, workflow, test logistics, standardization, and quality control. While the costs of sequencing consumables for GS are still more expensive than those for panel sequencing and ES, the laboratory process for generating GS data is simpler due to the lack of amplification and enrichment steps. This makes GS better suited for automation of sample preparation which translates into higher sample processing capacity with the same technical staff. The discontinuation of methods for CNV detection such as MLPA further reduces costs and time. Furthermore, panel sequencing requires continuous integration of novel genes with subsequent quality controls which can significantly prolong the diagnostic process. Also, because GS provides a nearly complete sequence of the patient’s genome, one can consider it at the DNA level an almost complete solution. Even considering the potential need for additional readouts such as transcriptome (see below) and epigenome data, or protein-based multi-omics data, the genome is well positioned to become the baseline of further analysis.

### Genome First and Ge-Med

Beginning in 2019, we performed a series of implementation studies via Genome First followed by Ge-Med which focus on the broader implications of genome-based medicine for patients, clinicians, and healthcare managers.

In the initial Genome First study (ClinicalTrial.gov-number: NCT03954652) we demonstrated the successful application of short-read GS (srGS) as a first-line routine diagnostic in a total of 450 patients across three indications including developmental delay and intellectual disability in children for which FraX, chromosomes, and microarray testing were negative (*n* = 200), childhood solid tumors^12^ (*n* = 100), and degenerative eye diseases^13^ (*n* = 150). We also addressed three key pillars that are critical to GS implementation: (1) Bridging the gap between theory and practice to overcome barriers (e.g., test logistics, workflow, turnaround time), (2) preparing clinicians and laboratories to integrate GS within their practice (e.g., test interpretation, education, and counseling), and (3) developing an understanding of how to effectively implement and scale GS in a diagnostic lab (e.g., automation, standardization, bioinformatics and data analysis beyond ES, and quality control).

**Fig. 2:**
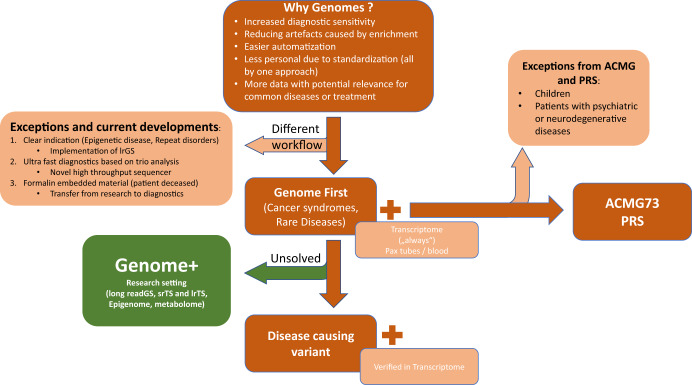
Overall approach and concept of Ge-Med. Genetic diagnostics (brown boxes) starts with whole genome analysis (srGS) and is associated with transcriptome sequencing (srTS). Data analysis for actionable genes and selected PRS are offered to the patients after genetic counseling. Exceptions from this Genome First approach are indicated (light brown). Unsolved patients may be enrolled into the Genome+ research concept allowing extended research NGS tools such as lrGS, extended srTS, epigenome, and metabolome analysis if patients give consent, fit research criteria, and request extended analysis (depicted in green).

Ge-Med was the next step to expand GS analysis to *all* rare diseases and familial cancer in our center (ClinicalTrial.gov-number: NCT04760522). It also allowed us to evaluate the feasibility of predictive genomics by analyzing actionable genes (https://www.ncbi.nlm.nih.gov/clinvar/docs/acmg/) and using polygenic risk scores (PRS) in patients with rare disease and familial cancer. Genetic risks can be provided on the basis of genome data for so-called actionable genes to direct prophylactic, preventive, or therapeutic interventions and also for population-based genetic risks for common diseases which can be partially defined by polygenic risk scores. Using the key implementation pillars identified in Genome First, we were able to closely examine the implementation of GS at scale in a routine clinical setting (Figs. 1 and 2). Importantly, the infrastructure and workflows that we developed to bring GS into diagnostics have been accredited by Germany’s national accreditation body (DAkkS). The Ge-Med concept has no limitation in numbers and is ongoing.

Here, we present the concept and conclusions from these studies, focusing on identifying key pain points and providing practical recommendations where possible.

## Results and lessons learned from Genome First and Ge-Med

### Test logistics, patients, and genetic counseling

**Fig. 3:**
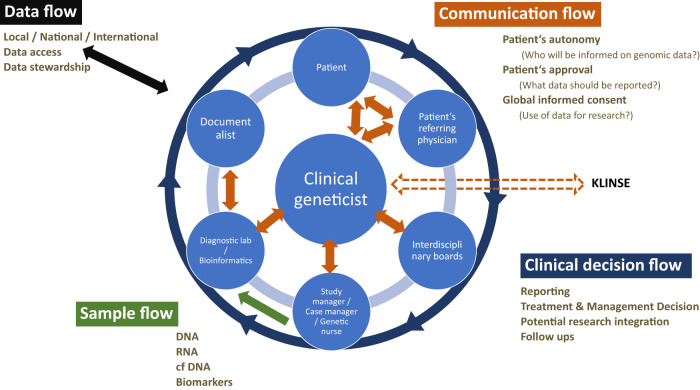
Complexity of the interaction of various individuals involved in the genome diagnostic process. Before diagnostic GS can be initiated, the full complexity and dimension of GS have to be communicated with the patient and the referring physician. In unclear and unsolved cases interdisciplinary boards decide whether to extend diagnostics to GS. In the process of sample collection, several caretakers need to be involved besides the clinical geneticist to ensure high-quality samples and complete documentation. Information and consent of patients have to be discussed at the end of the diagnostic process, as to where to store data, and who gets access to it. Using diagnostically generated data for research requires careful consideration of each stage of the diagnostic process. This model could support policymakers in developing novel diagnostic core centers ensuring widespread scientific use of diagnostic data in the healthcare system. KLINSE: Clinical Information Office on Rare Diseases (https://www.medizin.uni-tuebingen.de/de/das-klinikum/einrichtungen/zentren/zentrum-fuer-seltene-erkrankungen-zse/klinse).

Clinical implementation of diagnostic GS will require important procedural updates and modifications related to the patient care pathway (Fig. 3). For example, standard operating procedures (SOPs) for sample collection should be clearly defined (e.g., how to collect, type of blood vials, how to handle and potentially how to ship). Prior to extending diagnostics into research, ethical votes via the local medical ethical committees need to be granted. Patient involvement and counseling are required for extensive genome data analysis in a diagnostic setting. It is critically important to establish and maintain a level of trust and responsibility in the healthcare system in managing highly sensitive individual genome data.

Patient counseling beyond that of the current routine diagnostic procedure will need to include information related to the analysis and transmission of actionable gene data or even PRS, in addition to receiving informed consent (communication flow) to perform such analyses. Importantly, the extent of data analysis must be determined a priori using the following considerations: patient care infrastructure, access to disease experts, prevention programs for diseases with high genetic risks, and even to what extent health insurance will cover follow-up costs.

Given the complex integration of patients into different studies, it is important to establish the communication flow between the patient, physician/geneticist, other interacting clinicians, and the diagnostic institution. Transparent communication with the patient and the interacting clinicians on the data flow (where the data will be stored, who will have access, and under what conditions) is critical. There must also be a standard procedure for the information flow of the data (how to communicate results back to the patient), who will be allowed to obtain certain types of reports (healthcare professionals, relatives), and what clinical/preventive/management consequences can be drawn from the patient’s test results (Fig. 3).

Thus, adequate infrastructure is required to support a highly complex network of interacting specialists that includes multidisciplinary boards and case managers for the management of samples, clinical information, informed consent, and letters of referral. Additional headcount includes genetic nurses for professional sample collection for the different studies as well as a documentarist to manage the FAIR principles for data availability and sharing (Findable, Accessible, Interoperable, Reproducible)^14^. Health insurance companies often claim that because sequencing costs are falling, the reimbursement of the entire analysis should decrease as well. It is dually important for them to recognize the increasing complexity of this process as well as the potential of genome data to significantly impact patient care and inform disease prevention.

In our institute, we do not offer carrier status analysis as a routine procedure, but it may be offered in certain family situations (e.g., consanguinity of parents). Finally, there is a general agreement that genetic risk conditions without treatment options and without prevention should not be analyzed.

### Secondary findings (SF) and actionable genes (AG)

One example of the importance of communication between patients undergoing GS analysis and providers is the communication of secondary findings which should be embedded in the counseling process and include specific recommendations about follow-up medical check-ups.

Across a broad range of indications, the overall frequency of secondary findings is approximately 3%^15^. SF are informative in a few ways: (I) When a detailed retrospective anamnestic or pedigree analysis is performed or a clinical review reveals clinical manifestation in the person examined or symptomatic patients in the family, one can reasonably posit that GS did not reveal an SF per se but instead confirmed a previously not recognized diagnosis in the family (as relatively common for cancer syndromes); or (II) GS reveals a true SF without symptomatic patients in the family thus revealing true novelty for the new person at risk.

In both diagnostic and research settings, we have successfully established a process where patients can opt-in for analysis and reporting of SF. When genetic counseling is provided, information about the predictive nature of SF, their possible clinical consequences (preventive medical check-ups, preventive medical therapy, recommendation for lifestyle adaption), and their potential relevance to family members are discussed, equipping patients with information to help them decide whether to receive information on eventual SF or not. In our practice, we are not able to discuss each potential SF in detail; however, we inform about disease groups (e.g., cancer, cardiologically relevant genes, and actionable genes involved in metabolism). We also explain the difference between monogenic SF and a polygenic risk score. In compliance with national legal regulations (GenDG; https://www.gesetze-im-internet.de/gendg/index.html), patients can withdraw their consent as long as the results have not been reported. If SF is detected, a second report is provided independently of the original diagnostic analysis. This independent reporting has been established as it most frequently requires changes in patient management by a different medical specialist.

In general, our organization offers patients the option of requesting additional analysis of SF in AG and, most recently, for selected PRS (e.g., breast cancer, diabetes mellitus). Exceptions are analysis of AG/PRS in patients with psychiatric symptoms such as acute depression or schizophrenia, or in patients with severely progressed neurodegenerative diseases. In principle, these rules also apply to children, however, based on discussion with our ethical board, in selected cases, we inform the parents about their potential risk for a monogenic condition. Of course, genetic counseling and written informed consent are always required. Since 2016, we have applied the ACMG59 gene list, further developed by the German Network on Actionable Genes (GNAG; https://gfhev.de/de/ueber-uns/kommissionen.html). We acknowledge that the ACMG board of directors does not recommend global screening for variants in these genes^16^. With the extension of the gene list in 2021 (ACMG v3.0-v3.2)^17^, we adopted the recommended 73 genes or gene variants in the list of the reported AG. Overall, more than 90% of all patients in Ge-Med decided in favor of testing for all SF. With the precedent ACMG59 list, about 5% of all patients harbored pathogenic/likely pathogenic findings, a number which declined with the ACMG v3.0 recommendations despite including more genes (for instance, in addition to the ACMG59 list we reported also MUTYH heterozygosity and biallelic HFE variants whereas with ACMG v3.0 some common *HFE* mutations and MUTYH heterozygosity are not reported anymore). This is in good agreement with a recent study of about 58,000 individuals from Iceland who report that 4% of their population carry at least one actionable genotype^18^. In our clinic, variants of unknown clinical significance (VUS) are generally not reported as SF. We also highlight that (actionable) genes (such as *PMS2*) may not always be completely covered in a diagnostic sense (Box 1).

#### Polygenic risk scores

In addition to rare disease and familial cancer applications, genomic data can be used to generate a personalized risk assessment for common diseases as well as aid in predicting disease risk in healthy individuals. PRS have been developed to quantify the cumulative effect of multiple genomic loci on the predisposition to disease. To reveal this genetic architecture, large genome-wide association studies (GWAS) have been conducted and serve as a base for PRS development. An individual’s PRS is dependent on the risk alleles present which can be assessed by different technologies (SNP Array, GS). Currently, GWAS databases mostly consist of individuals of European ancestry and must be adapted when applied to individuals or populations of non-European ancestry^19^.

In European countries such as Germany, it is expected that the PRS for a certain disease will be normally distributed and stratified into risk groups from low to high. In 2018, Natarajan and coworkers demonstrated that PRS models could be derived from GS data^20^. In a large cohort of individuals with hypercholesterolemia, only 2% had monogenic mutations leading to hypercholesterolemia while 25% of patients with severe manifestation had a significantly increased PRS indicating the importance of PRS analysis for common diseases.

In Ge-Med, we sought to expand our GS service to well-established PRS for selected types of diseases to broaden the range of GS data analysis of common diseases. We calculated the PRS of 10 common diseases in a German cohort of 1000 unaffected individuals (suppl. Fig. 1). Up to 16% had at least one increased PRS, with two individuals (0.2%) even harboring four increased risks. It will be important to have ethical and clinical discussions about when and for whom these risks should be assessed as well as what diseases should be analyzed.

Our experience with Ge-Med has shown us that the full potential of disease-relevant data based on GS analysis can be achieved in a single workflow as one does not need to have an additional array of ES data for a patient. One also has flexibility in combining monogenic and polygenic risks, which can be easily and continuously adapted for novel PRS for various diseases. The potential future relevance of assessing PRS in the general population lies in risk prediction, stratified application of disease prevention programs, as well as informing diagnoses, predicting disease course, and potentially supporting treatment decisions^21^.

Over time, a better understanding of personal health and lifestyle data, in combination with environmental factors and genetic makeup, will allow for the most informed disease risk prediction. Considering this potential, it is clear that PRS will not be the only risk marker in this context but will rather be complemented by many clinical parameters. Thus, we include the body mass index (BMI) of individuals asking for their respective PRS in diabetes which will also be relevant in the extension of PRS to cardiovascular diseases.

It will also be important to define cut-offs for high-risk individuals, consider all risk factors over time, and assess the interactions between these risk factors. We are currently facing this challenge with female patients at high risk for breast cancer. We currently use CanRisk^22–24^ to integrate risk scores with other data such as family history, histology, and mutation profile, thus improving the risk estimate for breast cancer and facilitating recommendation of disease prevention programs according to the recommendations of the German Consortium for Hereditary Breast and Ovarian Cancer. For breast cancer, we have applied a risk score based on 313 variants^25^. We are also using an integrated risk model of BMI, age, and PRS for type-2 diabetes (T2D) and are developing a tailored disease prevention program in collaboration with our clinical partners for individuals with a high combined 10-year risk ( > 15%) or a high genetic risk, defined as PRS above percentile 90 together. While we have no data yet on if and how the transmission of this information to the patients does indeed influence lifestyle or medical management, we have taken the first steps to the implementation of PRS into a diagnostic process for two common diseases, breast cancer, and T2D.

#### Pharmacogenomics

Using the sequenced genome as a platform for pharmacogenomic (PGx) testing in clinical practice to understand how variations in the genome dictate the response to medications is another promising approach to leveraging the generated data for the benefit of the patient. We have not yet offered PGx analysis to our patients and/or relatives because this offering is not yet reimbursed by insurance and there is a lack of usage of the data among the medical community in Germany (except for dihydropyrimidine dehydrogenase deficiency) where testing is recommended in colorectal cancer before 5-FU treatment. This is likely to change, however, as countries begin developing guidance on how best to handle PGx. For example, The Netherlands has developed PGx guidelines and recommendations as part of the European Research consortium (U-PGx)^26^. The European Medicines Agency specifically addresses the utility of PGx to reduce medication side effects and to improve treatment response. Thus, PGx is likely to be integrated into the healthcare of other countries as it can lead to better outcomes for both individuals and healthcare providers through improved medication safety and efficacy and lowered medical costs.

Box 1 Pro: Beyond targeted diagnostics, clinical GS can be applied for risk prediction and disease prevention through the detection of actionable genes and PRS.

Con: Actionable genes and PRS are not part of a diagnostics contract in nearly all countries and are not requested by all patients. Thus, a combined diagnostic-research setting is required which needs a medical framework and cannot be an automatism for all situations and in each institution.

### Standardization

Importantly, the infrastructure and workflows that we developed to bring srGS into diagnostics have been accredited by Germany’s national accreditation body (DAkkS) according to DIN EN ISO 15189 as the formal structure for quality assurance. While laboratories performing NGS should be accredited according to EuroGenTest, in Germany, this is not mandatory under the requirements of the Genetic Diagnostics Act (GenDG). However, institutions carrying out genetic analyses for medical purposes have to meet quality requirements ensuring the suitability of qualified personnel, appropriate premises, documented procedures for handling of consumables, equipment and software, and pre-analytic measures. Implementation of NGS testing needs to cover quality assurance measures for wet laboratory, and data processing including primary, secondary, and tertiary analysis as well as defined pipeline quality control and validation cycles. Post-analytic measures comprise the requirements for the release of test results, standards for variant evaluation, data storage, and reporting of findings as well as quality assurance measures in terms of validating that the method is suitable for addressing a given medical question^27^. As for the latter point, we have benchmarked the ability of genome-based testing to detect single-nucleotide variants (SNVs) and small insertions and deletions (InDels) using the “genome in a bottle” (GIAB) sample NA12878. For the high-confidence regions, we observed a sensitivity of 99.6%/96.7%, positive predictive value (PPV) of 99.4%/99.4%, and genotyping accuracy of 99.9%/97.9% for SNVs and InDels, respectively (https://github.com/imgag/megSAP/blob/master/doc/performance.md). An in silico down-sampling analysis showed that the defined minimum diagnostic sensitivity of 95% (SNVs) and 90% (InDels) was still achieved with an average of 31x coverage. However, in a diagnostic context, we aimed for a minimum 38x mean coverage to avoid the need for resequencing due to variations in sample loading and to achieve a low number of ‘diagnostic gaps’ (regions with <20x coverage) in diagnostic core genes, e.g., for breast cancer (Box 2).

Box 2 We strongly recommend testing the diagnostic pipeline including bioinformatic tools using a reference sample. With clinical GS special attention should be given to the detection rate of InDels and repeat structures. We aim for a minimum of 38x mean coverage.

### Transcriptome sequencing (WTS)

Recent work has demonstrated that RNA sequencing can be beneficial for variant interpretation in rare diseases. Specifically, studies have reported that RNA sequencing can increase the diagnostic sensitivity up to 7.5%, while in the research setting it provides an additional 16.7% sensitivity with improved candidate gene resolution and the ability to evaluate splicing effects, copy number gain or loss, and regulatory variations all using GS data^28–30^. Lee and colleagues found that 18% of all genetic diagnoses returned required RNAseq to determine variant causality^31^.

It is important to note that the systematic integration of comprehensive RNA analysis in diagnostic reports is not yet fully established and current limitations in RNA analysis need to be addressed to enhance its utility as a diagnostic tool in human genetics.

First, tissue specificity for numerous genes may limit the ability to investigate the gene of interest, though others have shown that up to 90% of all genes may be covered by RNAseq^30,32^. In our experience, at the diagnostic level, we only detect about 60-70% of all OMIM genes in blood to support clinical GS (suppl. Fig. 2). For RNA analysis, we sequence at least 50 Mio clusters using polyA enrichment and sequencing as 2x100bp paired-end reads, which presents a sensitivity similar to a library preparation strategy that uses ribosomal and globin depletion (own unpublished data, and ref. ^33^). Deeper sequencing, however, may increase the number of OMIM genes detected by RNAseq based on peripheral blood.

Second, despite the availability of commercial products for RNA isolation and enrichment from blood samples, no method is cost-effective allowing for the investigation of RNA splicing and non-coding RNA. Depletion of ribosomal RNA results in a higher detection of introns, making the detection of potential splicing aberrations more challenging^33,34^. Enrichment of polyA RNA is sensitive to RNA degradation and results in a high and variable amount of globin transcripts, reducing the cost-effectiveness of the workflow^35,36^. The combination of polyA enrichment and ribosomal RNA depletion leads to an increase in intronic reads, and results in the loss of non-polyA transcripts.

Third, there is significant variability of transcriptome profiles depending on nutrition^37^, infection^38^, medication^39^, sex^40,41^, and age^42^. This variability may limit current global RNA data analysis in the diagnostic context. We have thus developed specific questionnaires for patients to learn at least about the most important potential RNA expression influencers (Suppl. Fig. 3). Diagnostic software for RNA analysis is under heavy development^43,44^. Most importantly, however, current algorithms tend to include predictions of the deleteriousness of variants, including tissue-specific gene expression prioritization^45^, indicating that several of the current limitations will be overcome soon with novel software analysis tools.

Even if global RNA analysis is not yet feasible for use as a first-line diagnostic, targeted analysis of predicted splice sites and the search for loss of allelic expression can be integrated into genome interpretation strategies and have become a valuable second readout. We have developed and implemented a novel workflow that allows us to perform both GS and RNA sequencing in a single-diagnostic process allowing far more comprehensive genomic data interpretation than GS alone. We have sequenced over 1,000 transcriptomes (human and cell lines) and developed specific questionnaires for the patients to gather information on vaccinations, infections, medication, and even special dietary requirements which all influence expression patterns but so far not yet specifically defined genes to reduce the complexity of individual transcriptome data. In addition to the 1,000 diagnostic RNAseq data mentioned above, we have generated an additional 5,000 in the research setting which could be used as a control set. We further generated diagnostic reports based on RNA analysis for DNA variants that have the potential to impact splicing, or in cases where only a single mutation has been identified in an individual with a putative autosomal recessive disease. Global, overall transcriptome analysis across all cohorts needs still to be done (Box 3) .

Box 3 Pro: WTS is useful for more precise analysis of predicted splice site alterations or when a second pathogenic variant is missing in the case of likely recessive diseases. In these cases, targeted WTS has the advantage of applying a routine protocol and is cheaper and faster than targeting a specific transcript of a gene, also considering the complexity of alternative spliced isoforms.

Con: Only about 50% of all OMIM genes are sufficiently expressed in blood to support clinical GS (suppl. Fig. 2). Gene transcription is highly sensitive to environmental factors and thus highly variable between humans. We administer questionnaires to patients to collect information on factors influencing expression. Due to this complexity, WTS should be used in selected cases and not as a primary diagnostic method. However, this is different in somatic cancer diagnostics for treatment decisions where we sequence WTS in parallel to ES/GS.

## Discussion and outlook

Considering the full potential of genome data in medicine for solving disease causes, optimizing targeted therapies (e.g., cancer), identifying genetic risk for common diseases, and providing PGx information for drug selection, there is great demand for embedding genomic data into a clinical care environment. This requires that the four main stakeholders of the healthcare system, patients and persons at risk, diagnostic and clinical partners, as well as health insurance companies, ask key questions to help inform decisions (Table 1).

**Table 1:** Key questions of healthcare partners in the diagnostic decision pathway

Healthcare partner	Key questions
Patients, Individuals at risk, relatives	“What would I like to know about my genes?” “Will I be able to handle this information?” “How will this information change my life?” “What are the benefits or harms and how likely are these?
Diagnostic partners	“What is feasible to analyze in a diagnostic setting with the currently available resources?” “How meaningful is the information based on the analytical data?” (e.g., PRS)
Clinical partners	“What are the consequences of test results?” “How do I communicate the data to the patients and to other potentially required specialists?” “What are the prediction and prevention programs?” “Is this covered by health insurance?”
Health insurance companies	“What are the predictive outcome measures and how can they be integrated into the healthcare system of the entire population?” “What are the costs that need to be spent and what costs can be saved?” “Where is the boundary between GS and its use as a clinical test versus research or biomarker discovery?”

As with any new concept in healthcare, stakeholder integration, each with different interests at the same time, can be challenging. The Ge-Med approach was developed to address the integration of key players and to demonstrate the first application of diagnostic reporting from monogenic to common diseases. Though we have not yet developed a cost model for spending and saving, we are currently engaging with health economists to support this effort.

Putting the diagnostic part into the center of the discussion requires additional considerations including (i) what is feasible to do in a routine setting, (ii) how solid is the scientific ground for the data, and (iii) what should and should not be reported.

### Feasibility

Although most analyses are in principle feasible, one must carefully consider the general computational effort and interpretation expertise underlying each diagnostic report. The implementation of decision support systems that can reliably detect, integrate, and interpret small variants, CNVs, structural variants, PRS, mobile element insertions, etc., and integrate RNA data in the context of DNA variant information is crucial for quality-assessed genome analysis in diagnostics and to reduce hands-on time of diagnostic personnel. Together with several other clinical partners we developed the open-source pipeline megSAP (https://github.com/imgag/megSAP) to address the points above.

In the European-funded Solve-RD project (https://solve-rd.eu), for instance, which is focused on using ES/GS for unsolved rare diseases, several bioinformatic working groups (Data Analysis Task Forces; DATF) reanalyze existing sequence data and Data Interpretation Task Forces (DITF) reevaluate this information in the context of the clinical phenotypes. This has led to an increase in diagnostic sensitivity by about 12% but requires integrating the expertise of numerous experts. A French initiative that combines genome, RNA, and epigenome analysis, found an increased diagnostic rate by about 33% compared to unsolved trio-exome analysis in a small cohort of 30 individuals with severe neurodevelopmental disorders^46^ but this may be due to the specific patient cohort analyzed. In our recent study on srGS of 1000 patients with eye diseases, we found pathogenic/likely pathogenic variants in non-coding genomic regions in about 13%^13^. For other disease cohorts, the increase of diagnostic sensitivity by GS compared to ES still needs to be determined. As Solve-RD was mainly working with exome data, one can anticipate that an entire network of experts such as the DATF will further improve diagnostic sensitivity based on genome data. However, expert networks as established in Solve-RD, operate in purely research settings and do not consider time constraints and regulations of the diagnostic body. Also, these research networks are commonly restricted to members receiving funding during a specified funding period, which limits their participation in a clinical diagnostic lab.

One can also anticipate that the development of artificial intelligence (AI) tools will undoubtedly improve existing bioinformatic analysis pipelines. Indeed, the first AI-based software algorithms like eDIVA, DeepPVP, or Fabric GEM^47–49^ are used to assist diagnostic processes and more will be entering routine clinical care and will enable recurrent and automated re-analysis of unsolved cases that integrate novel gene-disease-phenotype associations. At present, single-software solutions are not available that can delineate the cause of rare disease, while also interpreting PGx data and PRS for different common diseases. For example, in our laboratory, we must apply different software tools and check different databases for interpretation, all the while contending with the “need to know and do not want to know” requests of patients. Nevertheless, despite the steadily improving software tools, we still rely deeply on the expertise of the diagnostic team, how they interpret DNA variants in the context of the phenotype, what and how to report to the clinician, and of course on the amount of the data (numbers of clinical genomes) to be used as internal diagnostic reference source.

### Strength of the data

Point (ii) addresses the current scientific and medical knowledge of the relevance of genome data. Every month up to 50 novel genes or novel clinical phenotypes of known genes are being reported. In diagnostics, one must decide how solid these data are commonly describing only single families with one phenotype for a respective rare disease.

The success of moving from rare to common diseases as well as integration of PRS into diagnostics requires consideration and selection of a “best fit” respective risk score in a given population. For instance, the Polygenic Score catalog (PGS) lists 40 different PRS scores for breast cancer and different subtypes (https://www.pgscatalog.org/search/?q=breast+cancer, date of last accession: February 6^th^, 2023), though many more articles have been published on the application of PRS. Should we use a specific score for a woman with estrogen-receptor-positive breast cancer and use a more global PRS in unaffected women? In the presence of a family history, should a different score be applied and to what extent should we consider the ethnic background? Clearly, integrating all scores into our diagnostic pipelines and running all different constellations is not manageable. We have to make decisions also for practical reasons with all the limitations.

### Reporting

Point (iii) addresses the question of what should be reported. For PRS, while there is an overwhelming amount of data addressing high-risk PRS for disease prevention measures, there are only a few publications discussing the potential for low-risk profiles^49,50^. There is an ongoing discussion on variants of uncertain significance (VUS) reporting in different scenarios. In our practice, we do not report VUS in prenatal settings but do report VUS in the context of finding the disease cause if the phenotype fits. Late manifesting disorders with no prevention or treatment strategies should not be reported in our opinion. But how strict are our criteria and what is in the narrower sense “preventive”? There are well-established risk and prevention strategies for Alzheimer’s disease (AD) [https://wwwen.uni.lu/lcsb/news_events/dementia_research_and_prevention_in_luxembourg], but should we report monogenic and polygenic AD risks in diagnostics primarily aimed for other diseases and how well can they be integrated into our clinical healthcare systems?

#### The importance of clinical care networks

The implementation of genome sequencing into healthcare raises not only the discussion of extended diagnostics on the genetic and clinical counseling of patients or probands (pre-test phase), it also highlights the importance of well-established clinical networks for the care of patients/persons at risk and to further follow-up on these individuals (post-test phase). In well-informed societies in developed countries, the number of requests for genetic counseling is increasing faster than what can be provided by clinical service. At the same time, genetic counseling is complex and includes in-depth conversations with patients and/or parents that focus on (a) the disease and potential outcome of the testing, the relevance of diagnosis to the patient and his/her family, (b) the relevance of actionable genes, and c) the meaning of PRS. In addition, the clinical geneticist/genetic counselor must discuss which stakeholders have access to the report (e.g., doctors, family members) and explain the meaning of oftentimes multiple consent forms, each addressing different aspects of testing which can be challenging for patients to understand. We have encountered this challenge in Ge-Med where we needed to discuss the Ge-Med ethical form in addition to the legal obligation to provide information for genetic tests, and data protection information as well as the global informed consent of the faculty, and potentially some more specific ethical approvals for treatment and therapy.

In the case of positive findings, together with our clinical partners, we offer additional clinical sessions explaining the data, its relevance for the family, a potential change of management/treatment, follow-ups, and healthcare accessibility. Thus, including actionable findings and PRS in reporting increases the workload of the clinical geneticist, not only for the index person but also for family members. Despite this, disease prediction is a major step towards prevention or early detection and has a significant influence on the general health of the individual and on the long-term population.

In March 2013, the American College of Medical Genetics and Genomics (ACMG) released recommendations for reporting incidental findings in clinical exome and genome sequencing^51^ including a subset of conditions and genes or variants for which they expected a significant potential for preventing disease morbidity and mortality if identified in a presymptomatic context. Eventually, the ACMG established a “Secondary Findings Maintenance Working Group” (SFWG) to implement a process for updating this recommendation and has published updated lists of SF genes since. Conditions include cancer predisposition syndromes and cardiovascular or metabolism phenotypes. There is a general agreement that solely likely pathogenic or pathogenic variants should be reported.

However, ACMG recommendations for reporting of findings in AG are handled differently in various countries. Even within one country, there is no standard practice regarding what variants (gene lists) to report, and in what individuals. The ACMG updated several recommendations^17^ and many of them are being used as a blueprint to implement reporting of actionable findings. In Germany, we decided to have a Network on Actionable Genes (GNAG; https://gfhev.de/de/ueber-uns/kommissionen.html) which closely follows the ACMG recommendations. Nevertheless, actionable findings are not generally reported in Germany as these exceed the initial diagnostic request. The other “extreme” is that some institutions overrule the patients’ consent of not obtaining information on AG stating that information on a mutation in one of the cancer genes may save lives. In our institute, we decided to respect the patient’s decision of not receiving information about actionable variants if not explicitly requested. Furthermore, there are preliminary data that suggest that PRS modifies the risk of age at onset in mutation carriers for breast cancer in females^52–54^ and for breast and prostate cancer in males^55^. However, in our opinion data will have to be confirmed before integrating them into clinical care of unaffected women. The recently published predictions of manifestation of the contralateral breast in BRCA1/2 mutation carriers by the PRS^56,57^ is another strong argument to implement PRSs in addition to GS-based monogenic variant analysis in healthcare.

#### Long-read sequencing technologies and other -omics

With their ability to resolve some of the most challenging regions of the genome and to detect previously inaccessible structural variants, long-read next-generation sequencing technologies (lrNGS) have emerged as a powerful tool for the field of genomics^58,59^. lrNGS have been shown to be superior in differentiating active from pseudogenes, deciphering large complex repeat structures relevant for repeat expansion disorders, and defining the cis or trans status of mutations in one gene without the need to sequence the parents in parallel. With lrNGS technologies sequencing entire chromosomes “in one stretch” becomes feasible and will allow a new reference genome dataset such as T2T-CHM13 instead of the currently used GRCh38/hg38. However, T2T reference genomes will have to be generated in different populations before entering the clinic. It will be important to follow the sequencing quality, costs, and turnover capacity of lrNGS technologies (PacBio, ONT) and whether they increase diagnostic sensitivity (reviewed in ref. ^60^). Some lrNGS technologies (PacBio, ONT) are diagnostically interesting due to the “all in one” approach enabled by bioinformatic analysis of epigenetic genome modifications (reviewed in ref. ^61^). Independent of whether sr or lrNGS technologies will be the lead in GS diagnostics in the future, the more sequence data available in conjunction with detailed clinical, imaging, and biochemical lab data, the more precise genomic diagnostics and risk predictions will become.

Though lrGS was not implemented in the present set of studies, our laboratory is actively involved in several studies that will allow us to explore the potential of other technologies and methods, including lrNGS, that could help solve more rare disease cases. Specifically, we have explored the integration of diagnostics and research using two approaches. The first is to enroll patients into our project Genome+ (ClinicalTrials.gov ID: NCT04315727) allowing us to apply lrGS, long-read transcriptome sequencing (lrTS), as well as epigenome and multi-omics analysis in patients and families who provided informed consent (Fig. 2). The second is leveraging the European Reference Networks of Rare diseases (ERNs) to submit selected patients to the European-funded Network Solve-RD (https://solve-rd.eu/).

## Conclusions

Overall, though the integration of GS, transcriptome sequencing, SF, and PRS data into clinical care is challenging, it also represents a huge opportunity to provide patients and clinicians with the most comprehensive dataset to inform genomic predictive and preventive medicine. Individual safety and well-being should be the focus as the field moves towards extensive diagnostics; thus, it is critical that we define clinical care and management pathways for patients in these early stages. In our experience, establishing such a clinical care network has been met with great enthusiasm and open willingness of carefully counseled patients to get informed about their lifetime risks based on their genomic predisposition. The Ge-Med study allowed us to define the bottlenecks in this transition process from rare to common diseases starting with individual informed procedure, adapting data analysis pipelines and reporting, to enrolling high-risk individuals into a clinical care process demonstrating the great potential of sequencing genomes early in the diagnostic process of rare diseases and familial cancer syndromes. In Germany, GS will be feasible in rare disease centers for all patients with unsolved causes of a rare disease and of complicated tumor syndromes via the “Modellvorhaben 64e” (https://www.gesetze-im-internet.de/sgb_5/__64e.html) in 2024 covered by health insurance. The concept of providing clinical care and comprehensive genomic analysis from a single source may thus be used by other centers with a similar structure, for a fast implementation of omics-based personalized health concepts in medicine.

### Supplementary information

Additional supplementary information Final Revision

## Data Availability

https://github.com/imgag/megSAP.
