# Supplementary material for: Genomes in clinical care
Source: NPJ Genom Med. 2024 Mar 14;9:20. doi: 10.1038/s41525-024-00402-2 (PMC10940576; doi:10.1038/s41525-024-00402-2)
Supplement: Supplementary file 1 — Additional supplementary information Final Revision [file 41525_2024_402_MOESM1_ESM.pdf]

Data Analysis

Data analysis was performed via megSAP. Specifics and documentation are located at https://github.com/imgag/megSAP

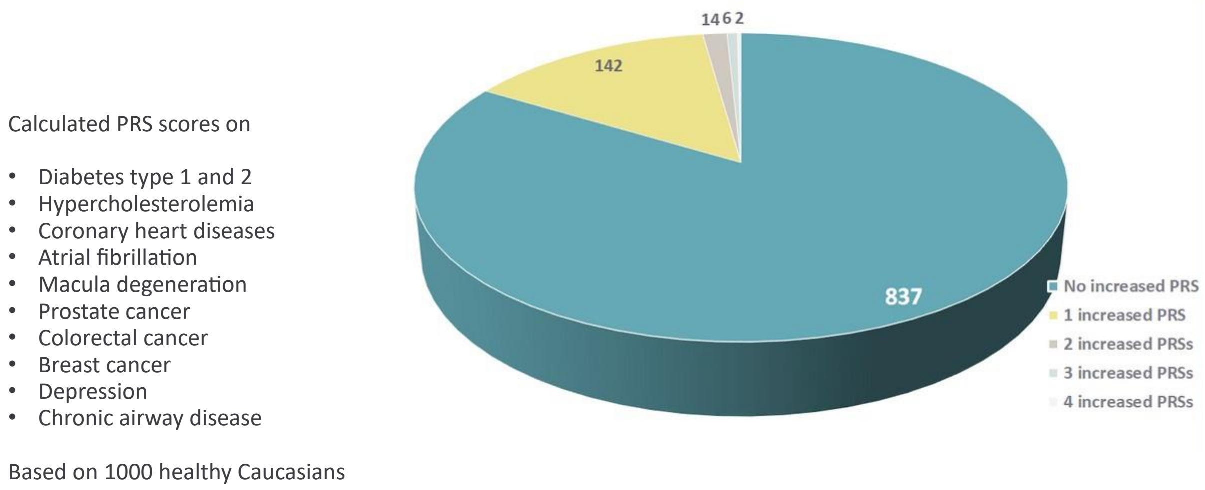

Suppl. Figure 1: Polygenic risks in a healthy population. Analysis of 10 different PRS in 1000 unrelated healthy Caucasians from our in-house data base indicating that more than 16% of the population are at higher risk for common polygenic diseases, some of them even for several diseases. The top 5% of the PRS scores were defined as "increased".
Applied PRS were used from the PGS catalogue as follows:
Diabetes mellitus Type 1: http://www.pgscatalog.org/publication/PGP000244/
Diabetes mellitus Type 2: http://www.pgscatalog.org/publication/PGP000100/
High cholesterol: http://www.pgscatalog.org/publication/PGP000244/
Coronary heart disease: http://www.pgscatalog.org/publication/PGP000100/
Atrial fibrillation: http://www.pgscatalog.org/publication/PGP000100/
Macular degeneration: http://www.pgscatalog.org/publication/PGP000263/
Prostate cancer: http://www.pgscatalog.org/publication/PGP000100/
Colorectal cancer: http://www.pgscatalog.org/publication/PGP000040/
Breast cancer: http://www.pgscatalog.org/publication/PGP000002/
Depression: http://www.pgscatalog.org/publication/PGP000068/
Chronic airway obstruction: http://www.pgscatalog.org/publication/PGP000244/

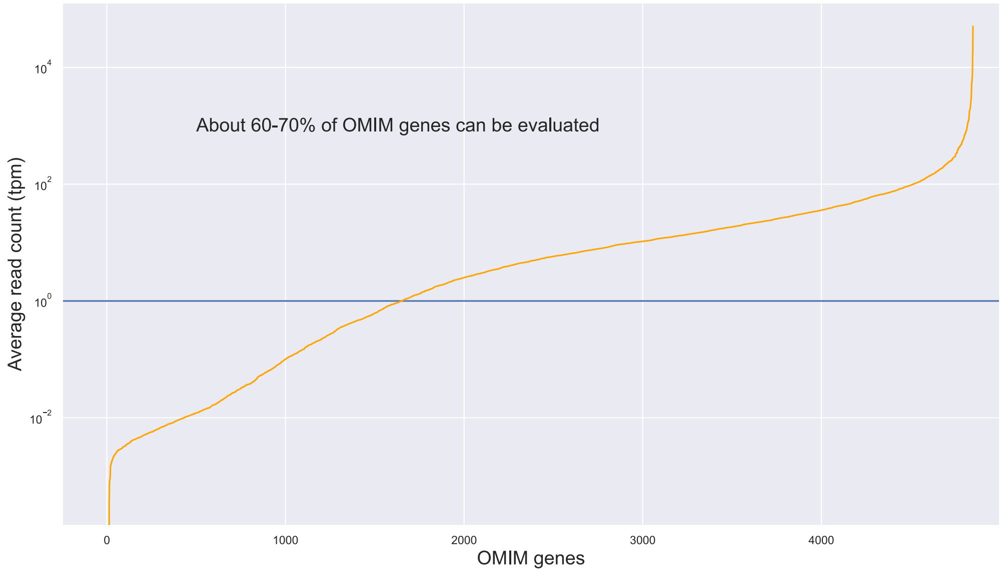

Suppl. Figure 2: Average gene expression of all OMIM genes in blood. For each OMIM gene, we counted the normalized number of reads using the tpm metric. (PAX-RNA samples), which pass QC (100M reads, housekeeping 20x coverage >65%) (Orange line) Blue line: Threshold expression for covered gene (>= 1 tpm)

Suppl. Figure 3: Patient information sheet associated with blood sampling for transcriptome analysis.

RNA-Seq data Sheet (English)

General information on the proband

Sticker with patient information here

Sample	☐ Pax- tube<br><br>☐ other RNA stabilizing tubes
Time of blood sampling (day and time)	
Other Tissue	
Infection last week?	☐ Yes ☐ No<br><br>If yes, please specify:
Vaccination last month	☐ Yes ☐ No<br><br>If yes, please specify:
Any drugs currently taken	☐ Yes ☐ No<br><br>If yes, please specify type and amount:
Any chronic diseases?	☐ Yes ☐ No<br><br>If yes, please specify:
Time of last meal	
Sport activity on the same day	☐ Yes ☐ No
Smoking	☐ Yes ☐ No<br><br>If yes, how frequent?

Patient‘s name
Signature

Medical doctor’s name
Signature
